# Clinical characteristics of different histologic types of breast cancer

**DOI:** 10.1038/sj.bjc.6602787

**Published:** 2005-09-20

**Authors:** C I Li, D J Uribe, J R Daling

**Affiliations:** 1 Division of Public Health Sciences, Fred Hutchinson Cancer Research Center, 1100 Fairview Ave. N., M4-C308, PO Box 19024, Seattle, WA 98109-1024, USA

**Keywords:** breast carcinoma, histology, SEER, stage, oestrogen receptor

## Abstract

Breast cancer is a heterogeneous disease, though little is known about some of its rarer forms, including certain histologic types. Using Surveillance, Epidemiology, and End Results Program data on 135 157 invasive breast cancer cases diagnosed from 1992 to 2001, relationships between nine histologic types of breast cancer and various tumour characteristics were assessed. Among women aged 50–89 years at diagnosis, lobular and ductal/lobular carcinoma cases were more likely to be diagnosed with stage III/IV, ⩾5.0 cm, and node-positive tumours compared to ductal carcinoma cases. Mucinous, comedo, tubular, and medullary carcinomas were less likely to present at an advanced stage. Lobular, ductal/lobular, mucinous, tubular, and papillary carcinomas were less likely, and comedo, medullary, and inflammatory carcinomas were more likely to be oestrogen receptor (ER) negative/progesterone receptor (PR) negative and high grade (notably, 68.2% of medullary carcinomas were ER−/PR− *vs* 19.3% of ductal carcinomas). In general, similar differences were observed among women diagnosed at age 30–49 years. Inflammatory carcinomas are associated with more aggressive tumour phenotypes, and mucinous, tubular, and papillary tumours are associated with less aggressive phenotypes. The histologic types of breast cancer studied here differ greatly in their clinical presentations, and the differences in their hormone receptor profiles and grades point to their likely different aetiologies.

Although breast cancer remains the most commonly diagnosed cancer among women in the United States (US) and worldwide, it is a heterogeneous disease. Breast cancer can be categorized in several ways, including based on its clinical features, its expression of tumour markers, and its histologic type. The two most common histologic types of invasive breast cancer are ductal and lobular carcinomas, accounting for approximately 75 and 15% of all cases in the US, respectively (Li *et al*, 2003a). Interest in lobular carcinoma in particular has recently been piqued by data indicating that incidence rates of lobular carcinoma are increasing more rapidly than are rates of ductal carcinoma in the US. Specifically, lobular rates have increased by 65% from 1987 to 1999, while rates of ductal carcinoma have increased only by 3% (Li *et al*, 2003a). Studies also suggest that lobular carcinomas are more likely than ductal carcinomas to be hormone receptor positive (Arpino *et al*, 2004; Korhonen *et al*, 2004). This difference may partly explain why seven studies have consistently observed that combined oestrogen and progestin postmenopausal hormone use is more strongly related to lobular carcinoma risk than to ductal carcinoma risk (Li *et al*, 2000, 2003b; Chen *et al*, 2002; Newcomb *et al*, 2002; Daling *et al*, 2003; Newcomer *et al*, 2003).

While several studies have now examined the clinical, pathologic, and epidemiologic differences between ductal and lobular carcinomas, much less is known about the rarer histologic types of breast cancer, including mucinous, tubular, comedo, inflammatory, medullary, and papillary carcinomas, which together account for about 10% of all cases. The purpose of this study is to characterise how rare histologic types of breast cancer differ in their stage, size, lymph node status, oestrogen receptor (ER)/PR status, and grade, utilizing data from 11 population-based tumour registries that participate in the Surveillance, Epidemiology, and End Results (SEER) Program. Evaluations of these differences are important for furthering our understanding of the nature of these tumours and may provide insight into the aetiologies and clinical features of different types of breast cancer.

## MATERIALS AND METHODS

Women diagnosed with a first primary invasive breast cancer at 30–89 years of age between January 1992 and December 2001 were identified through 11 population-based cancer registries in the US that participate in the National Cancer Institute's SEER Program. This age range was chosen because the vast majority of breast cancers diagnosed in the US occur among women in this age range (>98% based on the SEER data used here). Also, different aetiologic factors likely influence breast cancer occurrence among women younger than 30 or older than 90 years of age, but since too few women are diagnosed in these age ranges we could not assess them separately. The SEER registries that were used include those serving the states of Connecticut, Hawaii, Iowa, New Mexico, and Utah, and the urban areas surrounding Atlanta, GA; Detroit, MI; Los Angeles, CA; San Francisco-Oakland, CA; San Jose-Monterey, CA; and Seattle, WA. The standard for ascertainment of cases of cancer in the SEER registries is 98% (Surveillance Epidemiology and End Results, National Cancer Institute, 2005a). Individual patient medical records are the source of SEER data on patient and tumour characteristics. In general, the populations covered by SEER are representative of the whole US with regard to socioeconomic status and education level, though they include higher proportions of people living in urban areas and who are foreign born (Surveillance Epidemiology and End Results, National Cancer Institute, 2005b). Further operational details and methods used by the SEER Program are provided elsewhere (Young Jr *et al*, 1981).

In all, 199 721 women 30–89 years of age whose invasive breast cancer diagnosis was their first primary cancer diagnosis of any type were eligible for this study. Also, 539 women whose cancers were either not microscopically confirmed or were diagnosed only at autopsy were excluded. Women were grouped into the following nine histologic categories based on the ICD-O-3 code assigned to their tumours using an approach that has been previously published (Li *et al*, 2003c): ductal (defined using ICD-O code 8500), lobular (8520), ductal/lobular (8522), mucinous (8480), tubular (8211), comedocarcinoma (8501), inflammatory (8530), medullary (8510), and papillary (8050 and 8503). In addition, SEER's Extent of Disease (EOD) codes were used to identify additional inflammatory cases, since SEER EOD code 70 is used to define ‘inflammatory carcinoma, including diffuse (beyond that directly overlying the tumour) dermal lymphatic permeation or infiltration’. Hence, cases with a SEER EOD code of 70 were defined as inflammatory, regardless of the ICD-O-3 code they were assigned. The 10 167 women with other ICD-O codes, representing 5.1% of eligible subjects, were excluded from our analysis, leaving a total of 189 015 women. This group included women with diagnoses such as Paget's disease, because there were too few cases with these diagnoses to evaluate them separately in our analysis. In addition, we excluded 1116 subjects (5.9% of the total potentially eligible subjects) because they had an unknown or missing race/ethnicity. Since we were particularly interested in differences in ER and PR status by histology, we also excluded 52 742 cases (28.1% of the total potentially eligible subjects) because data on their ER and/or PR status were unknown. After these exclusions, 135 157 eligible women remained, including 102 463 ductal, 11 275 lobular, 9636 ductal/lobular, 3248 mucinous, 2222 comedo, 2095 inflammatory, 1983 tubular, 1617 medullary, and 618 papillary carcinoma cases.

In addition to histologic type, SEER registries also collect data on other tumour characteristics including AJCC stage at diagnosis, tumour size, lymph node status, ER and PR status, and tumour grade. Associations between the histologic type of breast cancer and each of these tumour characteristics were estimated using polytomous logistic regression (Begg and Gray, 1984), and based on likelihood ratio testing that compared models that included and excluded the tumour characteristic of interest, each of these tumour characteristics was statistically significant overall (*P*<0.00001 for each characteristic). We stratified our main analyses by age at diagnosis, evaluating the 100 028 women diagnosed at age 50–89 years and the 35 129 women diagnosed at age 30–49 years separately, because breast cancer risk factors, outcomes, and tumour characteristics differ by age, and particularly by menopausal status, and thus age is likely to be an important modifier of the associations assessed here (Bernstein, 1998). In the absence of information on menstrual history, 50 years of age has been shown to be a reasonable proxy for distinguishing postmenopausal from premenopausal women (Morabia and Flandre, 1992). Using Stata SE for Windows (Stata Corporation, College Station, TX, USA) statistical software, odds ratios (OR) and 95% confidence intervals (CI) were calculated. In all analyses, ductal carcinoma cases served as the reference histology group. All analyses were adjusted for age at diagnosis (continuous), year of diagnosis (continuous), SEER registry (categorical), and race/ethnicity (categorical). In addition, our analyses of stage, tumour size, and lymph node status were also adjusted for ER/PR status, our analyses of ER status were also adjusted for PR status, our analyses of PR status were also adjusted for ER status, and our analyses of tumour grade were also adjusted for stage and ER/PR status because each were hypothesized as potential confounders. Age at diagnosis (categorized as 30–49 years *vs* 50–89 years of age) was found to be an effect modifier of each of the relationships we assessed based on likelihood ratio testing, since *P*-values for interaction were all <0.05. In fact, all *P*-values for interaction were <0.00001, except those for tumour size (*P*=0.0014) and nodal status (*P*=0.0149).

Certain associations were not evaluated because the histopathologic definition of certain histologic types of breast cancer is dependent on the presence of particular clinical or pathologic features. Specifically, inflammatory carcinoma is defined by tumour emboli in dermal lymphatic vessels, and since >99% of inflammatory cases included in this study were stage III or stage IV and 98% were ⩾5.0 cm we did not evaluate stage and tumour size differences among inflammatory cases. Also, since tubular carcinomas are by definition well differentiated, and 99% of tubular cases in this study were grade 1 or 2, we did not evaluate tumour grade differences among tubular cases. Finally, since medullary carcinomas are characteristically poorly differentiated, and 90% of medullary cases in this study were grade 3 or 4, we did not evaluate tumour grade differences among medullary cases.

## RESULTS

Of the nine histologic types assessed, mucinous and papillary carcinoma cases had the oldest mean ages at diagnosis (65.8 and 65.7 years, respectively) and medullary carcinoma cases had the youngest mean age at diagnosis (52.8 years) (Table 1). While the number of ductal, lobular, ductal/lobular, mucinous, tubular, and inflammatory carcinoma cases increased over time, the number of comedo and medullary carcinoma cases declined and the number of papillary carcinoma cases held fairly constant. Variations in the racial/ethnic compositions of cases were also observed by histologic type.

Among women diagnosed with breast cancer at age 50–89 years of age, women with lobular, ductal/lobular, and inflammatory carcinoma were statistically more likely to be diagnosed with stage III/IV disease, while mucinous, tubular, comedo, and medullary carcinoma cases were less likely, compared to women with ductal carcinoma (Table 2). With respect to tumour size, women with lobular, ductal/lobular, and papillary carcinomas were more likely to be diagnosed with tumours that were 5.0 cm or larger (13.0, 7.5, and 9.5% of these women had tumours ⩾5.0 cm, respectively) compared to ductal carcinoma cases (5.6%), while tubular carcinoma cases were less likely (0.8%). Lobular, ductal/lobular, and inflammatory carcinoma cases also were more likely to be diagnosed with node-positive disease. Alternatively, mucinous, comedo, tubular, medullary, and papillary cases all were less likely to be diagnosed with node-positive disease. With respect to hormone receptor status, lobular, ductal/lobular, mucinous, tubular, and papillary carcinoma cases were 0.2–0.4-fold less likely to be diagnosed with ER−/PR− tumours, while comedo, medullary, and inflammatory cases were 1.7–11.6-fold more likely to be diagnosed with ER−/PR− tumours compared to ductal carcinoma cases. Finally, compared to ductal carcinoma cases, women with mucinous and papillary carcinomas tended to have lower-grade tumours, while comedo and inflammatory carcinoma cases tended to have higher-grade tumours.

Similar to the older women, among women diagnosed with breast cancer at age 30–49 years of age, those with lobular, and ductal/lobular carcinomas were more likely to be diagnosed with stage III/IV disease, while mucinous, comedo, tubular, and medullary carcinoma cases were less likely, compared to women with ductal carcinoma (Table 3). With respect to tumour size, women with lobular and ductal/lobular carcinomas were more likely to be diagnosed with tumours that were 5.0 cm or larger (18.1 and 12.1% of these women had tumours ⩾5.0 cm, respectively) compared to ductal carcinoma cases (8.6%), while tubular and medullary carcinoma cases were less likely (1.0 and 5.6%, respectively). Lobular, ductal/lobular, and inflammatory carcinoma cases also were more likely to be diagnosed with node-positive disease, and mucinous, comedo, tubular, and medullary cases were less likely, compared to ductal cases. In addition, lobular, ductal/lobular, mucinous, tubular, and papillary carcinoma cases were 0.2–0.5-fold less likely to be diagnosed with ER−/PR− tumours, while comedo, medullary, and inflammatory cases were 1.9–15.3-fold more likely to be diagnosed with ER−/PR− tumours compared to ductal carcinoma cases. Finally, compared to ductal carcinoma cases, women with lobular and mucinous carcinomas tended to have lower-grade tumours, while comedo and inflammatory carcinoma cases tended to have higher-grade tumours.

## DISCUSSION

Before interpreting the results of this study, it is important to acknowledge its limitations. First, the histologic categorizations used were based on diagnoses made by multiple pathologists in multiple institutions, and diagnostic criteria may vary somewhat by both individual pathologists and institutions, resulting in a certain degree of misclassification error. Studies evaluating the concordance between histologic classifications of breast tumours ascertained by SEER registries and those made through a centralized pathology review are needed to quantify the magnitude of this misclassification, as none have been reported in the literature. However, the misclassification of histologic type present in the SEER data is likely to be nondifferential, and as a result it may obscure differences but not lead to the identification of spurious differences. Also encouraging is that the proportions of cases in each histopathologic category were relatively similar across the 11 registries included in this study. One exception was that 10.9% of cases diagnosed in Los Angeles were ductal–lobular, while only 2.9% diagnosed in Hawaii were ductal–lobular. However, across the remaining nine registries, the proportions of cases that were ductal–lobular were relatively similar. Another concern is that we lacked information regarding certain potential confounders, including hormonal, reproductive, anthropometric, and lifestyle factors, that may be associated with both different histologic types of breast cancer and with the different clinical and pathologic tumour characteristics we evaluated. However, the strengths of this study are that it is large and population-based. Thus, it provides information on the tumour characteristics of different histologic types of breast cancer, some of which are quite rare, that are being observed in the general population.

Differences between various histologic types of breast cancer have been noted in prior studies. Two studies have explored the age distribution of different histologic types of breast cancer and have observed some striking differences. The first study by Stalsberg and Thomas (1993) reported that, while the relative frequency of ductal carcinoma is essentially constant by age, the frequencies of papillary and mucinous carcinomas tend to increase with age, the frequencies of medullary and inflammatory carcinomas tend to decrease with age, and the frequencies of lobular and tubular carcinomas increase until age 50, after which they remain fairly constant. A recent update of age-specific rates by histologic type observed three different age-rate patterns (Anderson *et al*, 2004). Specifically, rates of ductal, lobular, and tubular carcinomas were shown to rise sharply until age 50 and then rise more slowly, rates of papillary and mucinous carcinomas to rise steadily with age, and rates of medullary and inflammatory carcinoma to increase until age 50, after which they did not continue to rise. While this study also evaluated age-specific incidence rates by histologic type for ER+ and ER− tumours separately, it did not directly compare the distributions of ER status, or other clinical or tumour characteristics by histologic type.

In addition, risk of mortality has been observed to vary by histologic type as women with inflammatory breast cancers have an increased risk of mortality compared to women with other histologic types of breast cancer (Stierer *et al*, 1993; Anderson *et al*, 2003), while women with lobular, mucinous, comedo, tubular, medullary, and papillary carcinomas have lower risks of mortality compared to women with ductal carcinomas (Li *et al*, 2003c). However, few previous studies have evaluated differences in clinical and pathologic tumour characteristics by histologic type. With respect to ER/PR status, lobular, ductal/lobular, and mucinous carcinomas have been shown to be more likely to be ER+/PR+ compared to ductal carcinomas, similar to what we observed here (Desai *et al*, 2000; Arpino *et al*, 2004; Korhonen *et al*, 2004; Mathieu *et al*, 2004). However, studies have not evaluated ER/PR status in rarer histologic types of breast cancer, or evaluated differences in stage, tumour size, lymph node status, or grade by histologic type.

Here we observed several differences in these characteristics by histologic type, which only varied somewhat by age at diagnosis. The primary differences seen between our groups of largely postmenopausal women *vs* largely premenopausal women were differences in magnitudes rather than directions of risk. For example, among comedo carcinoma cases, the observed elevated risks of ER−/PR− tumours compared to ductal cases were more pronounced among the older women (OR=2.8) than among the younger women (OR=1.5). Exceptions to this were that lobular carcinomas diagnosed among younger women were less likely to be PR− compared to ductal cases, though this difference was not seen among the older women. In addition, older, but not younger, women with comedo carcinoma had an elevated risk of ER− tumours. Finally, the higher risk of tumours ⩾5.0 cm and the lower risk of node-positive tumours seen among older women with papillary carcinomas were not observed among younger women with papillary tumours. However, our ability to detect differences among papillary cases 30–49 years of age was limited by a relatively small sample size (*n*=91).

Despite these differences, among both age groups of women, mucinous, tubular, and papillary carcinomas generally had less aggressive phenotypes compared to ductal carcinoma cases, as they were each less likely to present at an advanced stage, to be node positive, to be hormone receptor negative, and to have a high grade. These features may explain why women with these tumours have relatively low risks of mortality (Stierer *et al*, 1993; Li *et al*, 2003c). In contrast, inflammatory carcinomas appear to have the most aggressive phenotype of any of the histologic types evaluated as these tumours were more likely to be node positive, to be hormone receptor negative, and to have a high grade. These findings are consistent with the poorer survival rates that women with these tumours experience, particularly for those with ER− inflammatory carcinoma (Anderson *et al*, 2003; Li *et al*, 2003c). Lobular, ductal/lobular, and comedo and medullary carcinomas had mixed phenotypes. Lobular and ductal/lobular tumours tended to be diagnosed at a more advanced stage and to be both >5.0 cm and node positive, but they were also much more likely to be hormone receptor positive. In contrast, comedo and medullary carcinomas were less likely to have an advanced stage at diagnosis and to be node positive, but more likely to be hormone receptor negative and to have a high grade. Interestingly, these characteristics appear to translate into all of these tumours having lower risks of mortality compared to ductal carcinoma cases, as we have reported previously (Li *et al*, 2003c).

Beyond differences in their histopathologic appearances, the results of this study suggest that different histologic types of breast cancer also differ substantially in their clinical and tumour characteristics. Interestingly, while inflammatory carcinomas are more likely to be characterized by poor clinical and pathologic tumour characteristics that do translate into poorer survival rates; lobular, ductal/lobular, comedo, and medullary carcinomas are characterized by a mix of tumour characteristics that are associated with both better and poorer prognoses, though previous data indicate that women with these tumours have lower risks of mortality than do women with ductal carcinomas (Li *et al*, 2003c). Thus, the prognostic importance and utility of the clinical and tumour characteristics evaluated here varies by histologic type. The differences in hormone receptor status and grade that we observed by histologic type may reflect the different aetiologies of these tumours. Further, the differences in stage, tumour size, and lymph node status observed here may reflect differences in the utility of screening approaches to detect different histologic types of cancer. For example, it is well known that lobular tumours are more difficult to detect with mammography compared to ductal tumours, and this is thought to be primarily due to the fact that lobular tumours tend to grow as linear strands or sheets of cancer cells rather than as more discrete masses, explaining why they are more likely to be diagnosed at a more advanced stage (Davis *et al*, 1979; Dixon *et al*, 1982; Silverstein *et al*, 1994; Yeatman *et al*, 1995). Thus, currently, available breast cancer screening tools appear to be relatively less or relatively more effective in detecting different histopathologic types of breast cancer.

## Figures and Tables

**Table 1:** Demographic characteristics of 139 310 women diagnosed with nine different histologic types of breast cancer

	**Ductal (*n*=102 463)**		**Lobular (*n*=11 275)**		**Ductal/lobular (*n*=9636)**		**Mucinous (*n*=3248)**		**Comedo (*n*=2222)**		**Inflammatory (*n*=2095)**		**Tubular (*n*=*1983*)**		**Medullary (*n*=1617)**		**Papillary (*n*=618)**	
	***n***	**%**	***n***	**%**	***n***	**%**	***n***	**%**	***n***	**%**	***n***	**%**	***n***	**%**	***n***	**%**	***n***	**%**
*Age at diagnosis*																		
30–39	6911	7	225	2	428	4	119	4	234	11	190	9	42	2	249	15	21	3
40–49	20754	20	1663	15	1849	19	399	12	616	28	518	25	344	17	497	31	70	11
50–59	24501	24	2534	23	2567	27	486	15	567	26	550	26	555	28	399	25	103	17
60–69	22505	22	2792	25	2173	23	731	23	436	20	388	19	536	27	272	17	146	24
70–79	19932	20	2820	25	1932	20	1005	31	287	13	308	15	397	20	153	10	177	29
80–89	7860	8	1241	11	687	7	508	16	82	4	141	7	109	6	47	3	101	16
Mean±s.d.	59.5±13.6		63.4±12.7		60.1±12.9		65.8±13.5		55.3±13.1		60.7±11.7		57.0±13.8		52.8±12.9		65.7±13.2	

*Diagnosis year*																		
1992–1993	17157	17	1791	16	1209	13	524	16	800	36	338	16	239	12	424	26	118	19
1994–1995	18445	18	1975	18	1377	14	571	18	553	25	338	16	331	17	342	21	133	22
1996–1997	20464	20	2203	20	1683	18	634	20	346	16	436	21	367	19	297	18	129	21
1998–1999	23315	23	2748	24	2388	25	725	22	288	13	482	23	516	26	283	18	118	19
2000–2001	23082	23	2558	23	2979	31	794	24	235	11	501	24	530	27	271	17	120	19

*Race/ethnicity*																		
Non-Hispanic white	78748	77	9605	85	7908	82	2467	76	1568	71	1518	73	1726	87	1004	62	434	70
Black	7927	8	555	5	563	6	199	6	246	11	253	12	71	4	287	18	69	11
Asian/Pacific Islander	8745	9	484	4	534	6	363	11	238	11	124	6	93	5	127	8	68	11
Hispanic white	6693	7	614	5	603	6	212	7	161	7	185	9	91	5	188	12	44	7
American Indian	350	0.3	17	0.2	28	0.3	7	0.2	9	0.4	15	0.7	2	0.1	11	0.7	3	0.5

s.d.=standard deviation.

**Table 2:** Relationship between breast cancer histology and tumour stage, ER/PR status, and grade among women diagnosed at 50–89 years of age^a^

	**Ductal** **(*n*=74 798)**	**Lobular (*n*=9387)**			**Ductal/lobular (*n*=7359)**			**Mucinous (*n*=2730)**			**Comedo (*n*=1372)**		
**Tumour characteristic**	**%**	**%**	**OR**	**95% CI**	**%**	**OR**	**95% CI**	**%**	**OR**	**95% CI**	**%**	**OR**	**95% CI**
*Stage* b													
I	53	44	1.0	ref	48	1.0	ref	68	1.0	ref	55	1.0	ref
II	38	43	1.5	1.4–1.6†	44	1.4	1.3–1.4†	28	0.6	0.6–0.7†	39	0.8	0.8–0.9†
III/IV	9	13	2.1	1.9–2.2†	8	1.2	1.1–1.3†	4	0.4	0.3–0.5†	6	0.6	0.4–0.7†

*Tumour size (cm)* b													
<2.0	61	48	1.0	ref	56	1.0	ref	65	1.0	ref	58	1.0	ref
2.0–4.9	34	39	1.6	1.6–1.7†	36	1.3	1.2–1.4†	30	0.9	0.8–1.0†	36	0.9	0.8–1.0
⩾5.0	6	13	3.5	3.3–3.8†	8	1.7	1.5–1.9†	5	1.0	0.8–1.2	7	0.9	0.7–1.1

*Lymph node status* b													
Negative	67	65	1.0	ref	62	1.0	ref	90	1.0	ref	72	1.0	ref
Positive	33	36	1.2	1.1–1.2†	38	1.3	1.2–1.3†	10	0.2	0.2–0.3†	28	0.7	0.6–0.8†

*ER* c													
ER+	78	92	1.0	ref	92	1.0	ref	96	1.0	ref	57	1.0	ref
ER−	22	8	0.3	0.2–0.3†	8	0.3	0.3–0.4†	5	0.2	0.2–0.3†	43	2.1	1.8–2.4†

*PR* c													
PR+	67	75	1.0	ref	78	1.0	ref	83	1.0	ref	49	1.0	ref
PR−	33	25	1.1	1.1–1.2†	22	0.9	0.8–0.9†	17	0.8	0.7–0.8†	51	1.3	1.2–1.5†

*ER/PR* c													
ER+/PR+	65	73	1.0	ref	77	1.0	ref	82	1.0	ref	44	1.0	ref
ER+/PR−	13	20	1.3	1.2–1.3†	15	1.0	0.9–1.0	13	0.8	0.7–0.9†	13	1.5	1.3–1.8†
ER-/PR+	2	2	0.7	0.6–0.8†	2	0.7	0.6–0.8†	1	0.3	0.2–0.5†	6	2.8	2.2–3.5†
ER−/PR−	19	6	0.3	0.2–0.3†	6	0.3	0.2–0.3†	4	0.2	0.1–0.2†	37	2.8	2.5–3.1†

*Grade* d													
1	18	23	1.0	ref	19	1.0	ref	58	1.0	ref	6	1.0	ref
2	44	53	0.9	0.8–0.9†	53	1.1	1.1–1.2†	35	0.3	0.2–0.3†	35	2.3	1.7–3.1†
3	36	21	0.5	0.4–0.5†	26	0.8	0.7–0.9†	6	0.1	0.1–0.1†	50	3.3	2.5–4.5†
4	2	3	1.1	0.9–1.3	2	1.2	1.0–1.5	1	0.1	0.04–0.2†	9	9.9	7.0–14.1†

	**Inflammatory (*n*=1387)**			**Tubular (*n*=1597)**			**Medullary (*n*=871)**			**Papillary (*n*=527)**		
	**%**	**OR**	**95% CI**	**%**	**OR**	**95% CI**	**%**	**OR**	**95% CI**	**%**	**OR**	**95% CI**
*Stage* b												
I	0.1	N/A^e^		92	1.0	ref	40	1.0	ref	57	1.0	ref
II	0.4			8	0.1	0.1–0.2†	56	1.5	1.3–1.7†	35	0.9	0.7–1.1
III/IV	99			1	0.1	0.03–0.1†	5	0.5	0.3–0.6†	8	0.9	0.6–1.2

*Tumour size, cm* b												
<2.0	2	N/A^e^		95	1.0	ref	42	1.0	ref	57	1.0	ref
2.0–4.9	9			4	0.1	0.1–0.1†	53	1.6	1.4–1.8†	33	1.1	0.9–1.3
⩾5.0	89			1	0.1	0.1–0.2†	6	0.8	0.6–1.1	10	1.9	1.4–2.5†

*Lymph node status* b												
Negative	9	1.0	ref	93	1.0	ref	71	1.0	ref	78	1.0	ref
Positive	91	19.3	15.2–24.6†	7	0.2	0.1–0.2†	29	0.7	0.6–0.8†	22	0.6	0.5–0.7†

*ER* c												
ER+	57	1.0	ref	95	1.0	ref	27	1.0	ref	88	1.0	ref
ER−	43	1.4	1.2–1.7†	5	0.2	0.2–0.3†	73	4.4	3.6–5.3†	12	0.8	0.6–1.1

*PR* c												
PR+	47	1.0	ref	81	1.0	ref	22	1.0	ref	82	1.0	ref
PR−	53	1.2	1.0–1.4†	19	1.0	0.8–1.1	78	2.6	2.1–3.2†	18	0.5	0.4–0.7†

*ER/PR* c												
ER+/PR+	42	1.0	ref	79	1.0	ref	17	1.0	ref	80	1.0	ref
ER+/PR−	15	1.3	1.1–1.6†	16	1.1	0.9–1.2	10	2.8	2.2–3.7†	8	0.5	0.3–0.6†
ER−/PR+	5	2.0	1.5–2.7†	2	0.7	0.5–1.1	4	5.5	3.8–7.9†	2	0.5	0.3–1.1
ER−/PR−	38	1.7	1.5–1.9†	3	0.2	0.1–0.2†	68	11.6	9.6–13.9†	10	0.4	0.3–0.6†

*Grade* d												
1	2	1.0	ref	86	N/A^e^		0	N/A^e^		40	1.0	ref
2	24	1.6	1.0–2.4†	13			11			42	0.4	0.3–0.5†
3	68	2.4	1.6–3.6†	1			78			17	0.2	0.1–0.3†
4	67	3.0	1.9–4.8†	0			11			2	0.4	0.2–0.8†

OR=odds ratio, CI=confidence interval, ER=oestrogen receptor, PR=progesterone receptor, N/A=not applicable.

† *P*<0.05.

a The reference histologic type for all analyses was ductal carcinoma.

b ORs are adjusted for age and year at diagnosis, cancer registry, race/ethnicity, and ER/PR status. Data on tumour size missing for 710 ductal, 130 lobular, 34 ductal/lobular, 10 mucinous, eight comedo, one tubular, three medullary, 94 inflammatory, and three papillary carcinomas. Data on lymph node status missing for 8051 ductal, 1001 lobular, 565 ductal/lobular, 464 mucinous, 136 comedo, 336 tubular, 43 medullary, 587 inflammatory, and 99 papillary carcinomas.

c ORs are adjusted for age and year at diagnosis, cancer registry, race/ethnicity, and stage. In addition, ORs for ER status are adjusted for PR status, and ORs for PR status are adjusted for ER status.

d ORs are adjusted for age and year at diagnosis, cancer registry, race/ethnicity, stage, and ER/PR status. Data on grade missing for 5732 ductal, 4149 lobular, 984 ductal/lobular, 981 mucinous, 367 comedo, 270 tubular, 321 medullary, 223 inflammatory, and 140 papillary carcinomas.

e These ORs were not calculated because almost all inflammatory carcinomas are stage III or IV and >5.0 cm in size, almost all tubular carcinomas are well differentiated, and almost all medullary carcinomas are poorly differentiated.

**Table 3:** Relationship between breast cancer histology and tumour stage, hormone receptor status, and grade among women diagnosed at 30–49 years of age^a^

	**Ductal (*n*=27 665)**	**Lobular (*n*=1888)**			**Ductal/lobular (*n*=2277)**			**Mucinous (*n*=518)**			**Comedo (*n*=850)**		
**Tumour characteristic**	**%**	**%**	**OR**	**95% CI**	**%**	**OR**	**95% CI**	**%**	**OR**	**95% CI**	**%**	**OR**	**95% CI**
*Stage* b													
I	40	36	1.0	ref	36	1.0	ref	61	1.0	ref	42	1.0	ref
II	50	48	1.2	1.1–1.4†	52	1.3	1.2–1.4†	35	0.5	0.4–0.6†	49	0.8	0.7–1.0†
III/IV	10	16	2.4	2.1–2.7†	12	1.7	1.5–1.9†	4	0.3	0.2–0.5†	9	0.7	0.6–1.0†

*Tumour size (cm)* b													
<2.0	49	43	1.0	ref	48	1.0	ref	59	1.0	ref	48	1.0	ref
2.0–4.9	42	39	1.3	1.2–1.4†	40	1.1	1.0–1.2†	34	0.8	0.6–0.9†	41	0.9	0.8–1.0
⩾5.0	9	18	3.4	2.9–3.9†	12	1.8	1.6–2.2†	7	0.8	0.5–1.1	12	1.2	0.9–1.5

*Lymph node status* b													
Negative	57	54	1.0	ref	48	1.0	ref	85	1.0	ref	60	1.0	ref
Positive	43	46	1.2	1.1–1.3†	52	1.4	1.3–1.6†	15	0.2	0.2–0.3†	40	0.8	0.7–1.0†

*ER* c													
ER+	66	89	1.0	ref	87	1.0	ref	91	1.0	ref	56	1.0	ref
ER−	34	11	0.4	0.3–0.5†	13	0.4	0.4–0.5†	9	0.2	0.1–0.3†	44	1.2	1.0–1.5

*PR* c													
PR+	63	85	1.0	ref	83	1.0	ref	81	1.0	ref	54	1.0	ref
PR−	38	15	0.5	0.5–0.6†	17	0.6	0.5–0.7†	19	1.1	0.8–1.4	46	1.3	1.0–1.5†

*ER/PR* c													
ER+/PR+	58	81	1.0	ref	80	1.0	ref	79	1.0	ref	46	1.0	ref
ER+/PR−	8	8	0.7	0.6–0.8†	8	0.6	0.5–0.8†	12	1.1	0.9–1.5	11	1.6	1.2–2.0†
ER−/PR+	5	4	0.6	0.5–0.8†	3	0.5	0.4–0.7†	2	0.3	0.2–0.6†	8	1.6	1.2–2.1†
ER−/PR−	29	7	0.2	0.1–0.2†	10	0.2	0.2–0.3†	7	0.2	0.1–0.3†	36	1.5	1.3–1.7†

*Grade* d													
1	10	19	1.0	ref	14	1.0	ref	45	1.0	ref	4	1.0	ref
2	37	55	0.8	0.7–0.9†	51	1.0	0.9–1.1	41	0.3	0.2–0.4†	28	1.7	1.1–2.6†
3	50	22	0.3	0.3–0.4†	32	0.6	0.5–0.7†	13	0.1	0.1–0.1†	57	2.5	1.7–3.8†
4	3	3	0.7	0.5–1.0	3	0.9	0.7–1.2	1	0.1	0.03–0.3†	11	7.2	4.5–11.7†

	**Inflammatory (*n*=708)**			**Tubular (*n*=386)**			**Medullary (*n*=746)**			**Papillary (*n*=91)**		
	**%**	**OR**	**95% CI**	**%**	**OR**	**95% CI**	**%**	**OR**	**95% CI**	**%**	**OR**	**95% CI**
*Stage* b												
I	0	N/A^e^		87	1.0	ref	33	1.0	ref	42	1.0	ref
II	1			12	0.1	0.1–0.2†	62	1.1	0.9–1.3	50	0.9	0.6–1.5
III/IV	99			1	0.03	0.01–0.1†	4	0.3	0.2–0.5†	9	0.8	0.4–1.8

*Tumour size (cm)* b												
<2.0	2	N/A^e^		92	1.0	ref	34	1.0	ref	52	ref	
2.0–4.9	7			7	0.1	0.1–0.2†	60	1.4	1.2–1.7†	36	0.8	
⩾5.0	91			1	0.1	0.03–0.2†	6	0.6	0.4–0.8†	12	1.4	

*Lymph node status* b												
Negative	9	1.0	ref	91	1.0	ref	69	1.0	ref	59	1.0	ref
Positive	91	12.7	9.0–17.8†	9	0.1	0.1–0.2†	31	0.6	0.5–0.7†	41	0.9	0.6–1.4

*ER* c												
ER+	43	1.0	ref	93	1.0	ref	14	1.0	ref	77	1.0	ref
ER−	57	1.7	1.4–2.2†	8	0.2	0.2–0.4†	86	5.8	4.4–7.6†	23	0.7	0.4–1.4

*PR* c												
PR+	41	1.0	ref	85	1.0	ref	16	1.0	ref	75	1.0	ref
PR−	60	1.1	0.9–1.4	15	0.8	0.6–1.1	84	2.5	2.0–3.2†	25	0.7	0.4–1.3

*ER/PR* c												
ER+/PR+	34	1.0	ref	82	1.0	ref	10	1.0	ref	70	1.0	ref
ER+/PR−	9	1.2	0.9–1.6	10	1.0	0.7–1.4	4	3.2	2.1–4.8†	7	0.6	0.3–1.5
ER-/PR+	7	1.9	1.3–2.7†	3	0.5	0.3–1.0†	7	7.1	4.9–10.3†	4	0.7	0.2–1.9
ER−/PR−	50	1.9	1.6–2.3†	5	0.2	0.1–0.3†	80	15.3	11.9–19.6†	19	0.5	0.3–0.9†

*Grade* d												
1	1	1.0	ref	87	N/A^e^		1	N/A^e^		22	1.0	ref
2	20	2.2	1.0–4.9†	12			6			49	0.6	0.3–1.1
3	72	2.7	1.3–5.9†	1			81			23	0.2	0.1–0.5†
4	6	2.8	1.2–6.7†	0			1			6	0.9	0.3–2.9

OR=odds ratio, CI=confidence interval, ER=oestrogen receptor, PR=progesterone receptor, N/A=not applicable.

† *P*<0.05.

a The reference histologic type for all analyses was ductal carcinoma.

b ORs are adjusted for age and year at diagnosis, cancer registry, race/ethnicity, and ER/PR status. Data on tumour size missing for 209 ductal, 24 lobular, 17 ductal/lobular, three mucinous, five comedo, 36 inflammatory, and one papillary carcinomas. Data on lymph node status missing for 1242 ductal, 118 lobular, 87 ductal/lobular, 28 mucinous, 54 comedo, 26 tubular, 17 medullary, 304 inflammatory, and six papillary carcinomas.

c ORs are adjusted for age and year at diagnosis, cancer registry, race/ethnicity, and stage. In addition, ORs for ER status are adjusted for PR status, and ORs for PR status are adjusted for ER status.

d ORs are adjusted for age and year at diagnosis, cancer registry, race/ethnicity, stage, and ER/PR status. Data on grade missing for 1732 ductal, 794 lobular, 289 ductal/lobular, 198 mucinous, 215 comedo, 75 tubular, 251 medullary, 102 inflammatory, and 22 papillary carcinomas.

e These ORs were not calculated because almost all inflammatory carcinomas are stage III or IV and greater than 5.0 cm in size, almost all tubular carcinomas are well differentiated, and almost all medullary carcinomas are poorly differentiated.
